# Expression of miRNAs and Their Cooperative Regulation of the Pathophysiology in Traumatic Brain Injury

**DOI:** 10.1371/journal.pone.0039357

**Published:** 2012-06-22

**Authors:** Zhonghua Hu, Danni Yu, Camila Almeida-Suhett, Kang Tu, Ann M. Marini, Lee Eiden, Maria F. Braga, Jun Zhu, Zheng Li

**Affiliations:** 1 Unit on Synapse Development and Plasticity, National Institute of Mental Health, National Institutes of Health, Bethesda, Maryland, United States of America; 2 Laboratory of Cellular and Molecular Regulation, National Institute of Mental Health, National Institutes of Health, Bethesda, Maryland, United States of America; 3 Genetics and Developmental Biology Center, National Heart Lung and Blood Institute, National Institutes of Health, Bethesda, Maryland, United States of America; 4 Department of Statistics, Purdue University, West Lafayette, Indiana, United States of America; 5 Department of Anatomy, Physiology and Genetics, School of Medicine, Uniformed Services University of the Health Sciences, Bethesda, Maryland, United States of America; 6 Department of Neurology, School of Medicine, Uniformed Services University of the Health Sciences, Bethesda, Maryland, United States of America; University of North Dakota, United States of America

## Abstract

Traumatic brain injury (TBI) is a leading cause of injury-related death and disability worldwide. Effective treatment for TBI is limited and many TBI patients suffer from neuropsychiatric sequelae. The molecular and cellular mechanisms underlying the neuronal damage and impairment of mental abilities following TBI are largely unknown. Here we used the next generation sequencing platform to delineate miRNA transcriptome changes in the hippocampus at 24 hours and 7 days following TBI in the rat controlled cortical impact injury (CCI) model, and developed a bioinformatic analysis to identify cellular activities that are regulated by miRNAs differentially expressed in the CCI brains. The results of our study indicate that distinct sets of miRNAs are regulated at different post-traumatic times, and suggest that multiple miRNA species cooperatively regulate cellular pathways for the pathological changes and management of brain injury. The distinctive miRNAs expression profiles at different post-CCI times may be used as molecular signatures to assess TBI progression. In addition to known pathophysiological changes, our study identifies many other cellular pathways that are subjected to modification by differentially expressed miRNAs in TBI brains. These pathways can potentially be targeted for development of novel TBI treatment.

## Introduction

Traumatic brain injury (TBI) is an insult to the brain from the application of external physical force that leads to temporary or permanent structural and functional impairment of the brain. TBI is a leading cause of injury-related death and disability [1]. Around 1.7 million people sustain a TBI in the U.S. annually and 53,000 of them die from TBI-related injuries [2], [3]. In TBI survivors, neuropsychiatric abnormalities, such as cognitive deficits, emotional and behavioral problems are common and contribute substantially to post-TBI disabilities [4]. The hippocampus is an essential brain region for cognition and emotion [5], and is vulnerable to TBI. TBI-related hippocampal damages, such as cell loss, disturbed neural circuits and impaired synaptic transmission and synaptic plasticity, are major pathophysiological changes in TBI [6], [7], [8], and are expected to lead to neuropsychiatric symptoms. However, the molecular and cellular mechanisms responsible for hippocampal damage and recovery following TBI are poorly understood. The structural and functional modification of the brain entails gene expression changes. In fact, studies in both humans and animal models of TBI show that gene expression is altered by TBI in multiple brain regions [9], [10], [11], [12], but the molecular mechanisms underlying TBI-induced gene expression changes are unclear.

MicroRNAs (miRNAs) are a class of small non-coding RNAs which repress gene expression at the post-transcriptional level by translational inhibition and/or promoting mRNA degradation through binding of the miRNA seed regions (usually nucleotides 2–8 at the 5′ end) to their binding sites in the 3′ untranslated region (3′ UTR) of the target mRNAs [13]. Each miRNA can potentially target up to hundreds of protein-coding transcripts due to imperfect base pairing between miRNAs and their targets. Hence, miRNAs exert pleiotropic effects on gene expression and regulate diverse biological processes, such as cell proliferation and differentiation and apoptosis [14]. Over 1000 miRNAs have been identified in mammals and hundreds of them are expressed in the brain [15], [16]. miRNAs are crucial for proper brain functions. Loss of miRNAs in mice due to deficient expression of Dicer and DGCR8, which are two miRNA processing molecules, causes changes in synaptic protein expression, synaptic transmission, dendritic spine morphology, learning and memory [17], [18]. It has been reported that TBI alters miRNA expression in the hippocampus [19]. Because changed miRNA expression is predicted to influence their target mRNA expression, it is possible that miRNAs play a role in regulating gene expression in TBI. Hence, a comprehensive analysis of miRNA expression profiles and identification of miRNAs modulated in TBI are essential to construct the signaling network responsible for TBI-induced gene expression changes.

Animal models are important tools for studying TBI. The lateral fluid percussion injury (FPI) model and the controlled cortical impact injury (CCI) model are two widely used animal models of focal TBI. FPI is delivered through a pressurized pulse of saline solution created by a piston [20]. CCI is produced by a rigid impactor which delivers a mechanical energy to the intact dura. The parameters of CCI, such as impact velocity and depth, can be fine-tuned by adjusting the settings of the impactor, allowing injury severity to be precisely controlled [21].

Here, by employing the next generation sequencing platform, we analyze the miRNA transcriptomes in the rat hippocampus at 24 hours and 7 days after TBI in the CCI model. Deep sequencing provides superb sensitivity, quantifiability and throughput over microarray and quantitative RT-PCR assays for miRNAs. We show that distinct miRNAs are up- or down-regulated at the two post-CCI time points. To decipher the biological implication of altered miRNA expression, we design a miRNA-gene-GO enrichment analysis to identify biological functions that are regulated by the target genes of changed miRNAs. We find that different gene ontology (GO) terms are targeted by the miRNAs altered in expression at the early and late phase of TBI. Intriguingly, the biological processes associated with these GO terms are consistent with the characteristic post-CCI pathological, physiological and structural changes. These findings indicate that miRNA expression profile changes are a distinctive molecular signature of post-TBI brains, and regulation of gene expression by miRNAs might be an important component of the cellular programs used by the injured brain to modulate gene expression for management and repair of injury-related cell damages.

## Results

### miRNA Transcriptome Changes in the Hippocampus Following CCI Injury

To interrogate the effect of TBI on miRNA expression, we used Illumina sequencing to analyze CCI-induced miRNA transcriptome changes. Male rats aged 60 days were sham operated or subjected to CCI (3–4 rats for each condition), and euthanized for collection of brain tissues at 24 hours and 7 days after CCI. RNA was extracted from the dorsal hippocampus, which corresponds to the human posterior hippocampus and is involved in learning and memory [5], and assessed for RNA quality by measuring the RNA integrity number (RIN) (Table S1). Sequencing libraries were prepared from miRNAs extracted from the dorsal hippocampus on the injured side of the brain (Figure S1A) and sequenced using an Illumina GAII analyzer. 0.64–1.0 million quality-filtered reads (see deep-sequencing data analysis in Materials and Methods) were obtained from each sample, 89–93% of them were mapped to mature rat miRNAs, and 328 mature rat miRNAs were identified from the total of 12,520,384 mapped reads from 15 rats (Table S1). The number of miRNAs detected by our assay is in line with a recent report that 294 miRNAs are detected in the mouse brain [15].

**Figure 1:**
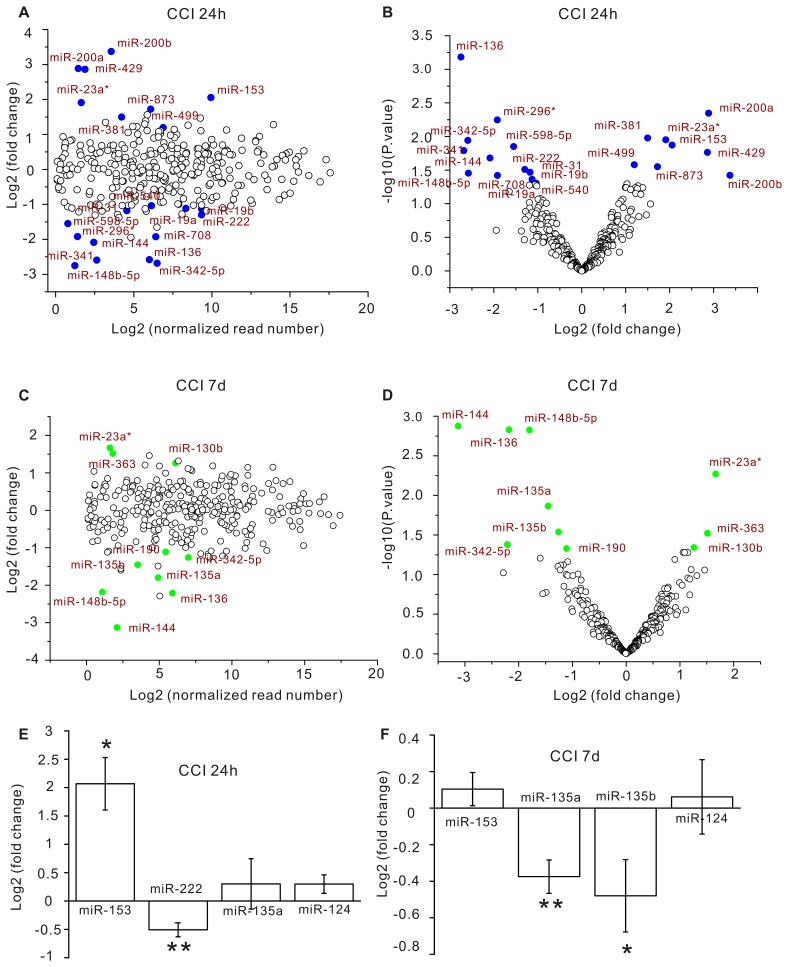
miRNA expression changes in the CCI brains. The hippocampus on the operated side of the brain was removed for miRNA expression profiling by deep sequencing at 24 hours (A, B) and 7 days (C, D) after CCI. Each circle represents a miRNA species. (A, C) The fold change (on log2 scale) of each miRNA species in CCI rats versus sham controls is plotted against its normalized read number (on log2 scale). (B, D) The p-value (on log10 scale) of each miRNA species is plotted against its fold change (on log2 scale). Blue and green dots are miRNAs that have p-values less than 0.05. (E, F) The fold change (on log2 scale) of miRNAs analyzed by qRT-PCR. Data are presented as mean ± s.e.m. Student’s t test was used for statistical analysis. * p<0.05; ** p<0.01.

The normalized tag counts of individual miRNAs in each library were used to assess their relative abundance. The fold change and p-values of miRNA expression changes detected by deep sequencing were illustrated in Figure 1, 2. In the MA plots of Figure 1A, C, the fold changes were randomly distributed around zero, but independent on the relative expression levels of individual miRNAs. This observation indicates that our deep sequencing was unbiased on miRNA abundance. The p-values of both the 24-hour and 7-day datasets were uniformly distributed between 0 and 1 (Figure S1B, C), thus the null hypothesis was true and the assumption of t-test was satisfied. It is worth noting that all miRNAs with p-values less than 0.05 had absolute fold changes greater than 1 (Figure 1B, D). The correlation between p-value and fold change indicates that the statistical significance and the biological significance of the sequencing result are consistent.

**Figure 2:**
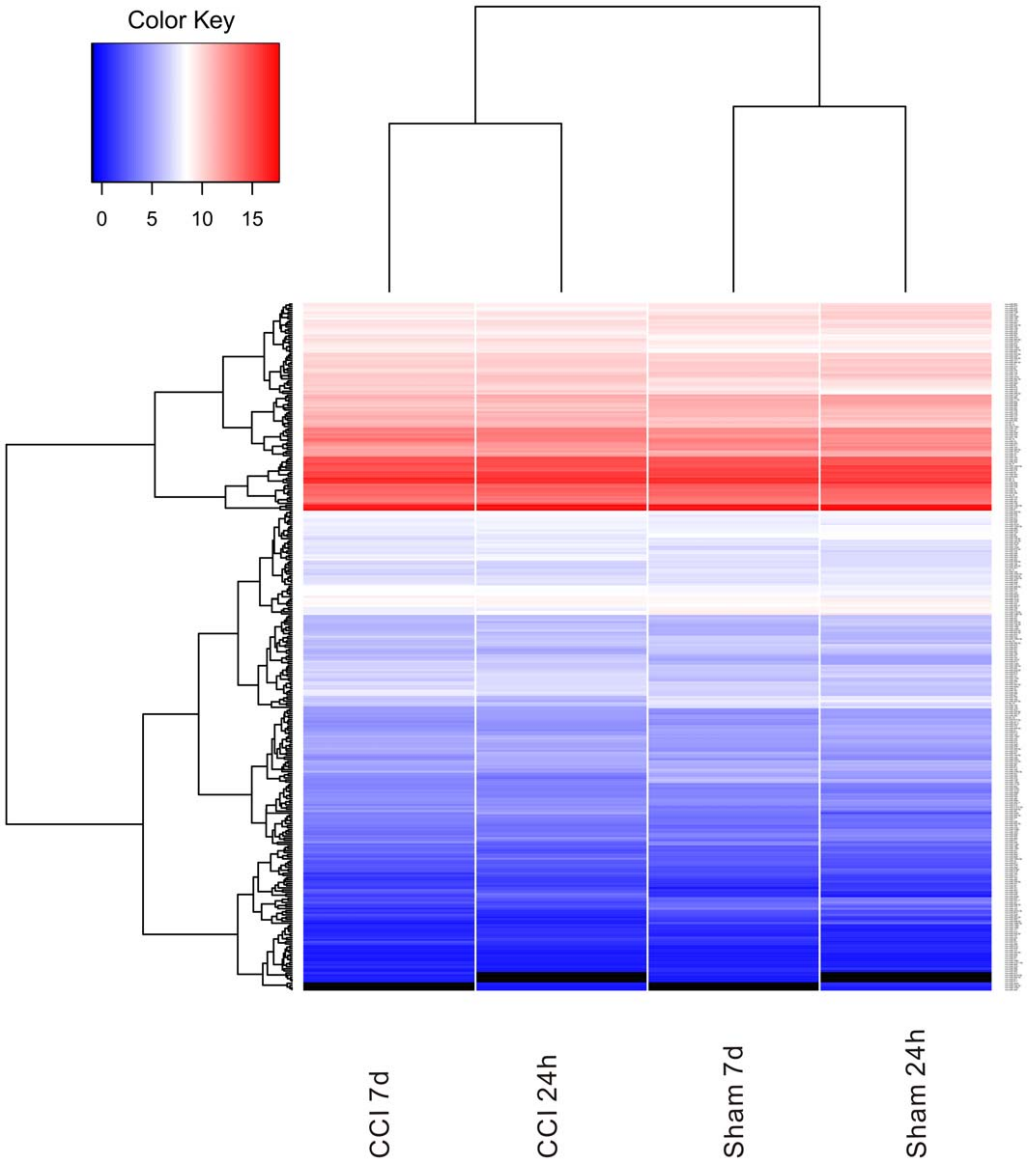
Heat map of miRNAs analyzed in the CCI brain. Heat map of mean signal intensities following normalization for all miRNAs detected in the CCI and sham control hippocampi by deep sequencing.

To validate the results of deep sequencing with an independent assay, we used quantitative reverse transcription polymerase chain reaction (qRT-PCR) to measure the expression change of miR-124, miR-135, miR-153 and miR-222. As shown in Figure 1E, F, miR-153 expression was elevated and miR-222 expression was decreased at 24 hours post-CCI; expression of both miR-135a and miR-135b was reduced at 7 days post-CCI; miR-124 expression was comparable in control and CCI samples at both the 24-hour and 7-day time points. These qRT-PCR results were consistent with the findings from deep sequencing (Figure 1A–D).

By using deep-sequencing, we found that 13 miRNAs were down-regulated and 8 miRNAs were up-regulated at 24 hours after CCI (p<0.05, Figure 1A, B). Among the 21 miRNAs altered at 24 hours, 5 (miR-144, miR-136, miR-148b-5p, miR-342-5p, miR-23a*) showed persistent changes by 7 days after CCI (Figure 1C, D). The number of changed miRNAs was reduced to 10 (7 down-regulated and 3 up-regulated) at 7 days post-CCI (Figure 1C, D). These results show that up to 6% of miRNAs expressed in the hippocampus are changed following CCI injury. The finding that overlapping but distinct sets of miRNAs are changed at 24 hours and 7 days post-CCI implicates that the miRNA transcriptome change is a prominent molecular signature of the injured brain for different post-injury times.

### miRNA-gene-GO Enrichment Analysis of miRNAs Altered by CCI Injury

The cellular and physiological effects of altered miRNA expression are mainly mediated by their regulation of target mRNAs. Many miRNAs are predicted to have hundreds of mRNA targets, and each mRNA usually has binding sites for multiple miRNA species. While the repressive effect of an individual miRNA species on its target expression can be moderate, the combinatorial control of a gene by multiple miRNA species that bind to the same mRNA target, and the concurrent action of one miRNA species on several target genes belonging to the same signaling pathway are expected to regulate gene expression and biological functions in a more robust and fine-tuned manner [22].

To test the possibility that multiple miRNA species function cooperatively in TBI, we developed a miRNA-gene-GO enrichment analysis to computationally examine the potential effects of miRNA expression changes in CCI. The effect size of miRNA expression change on its target gene expression and cellular activities varies greatly among miRNAs, and is determined by the fold change of the miRNAs. Because the absolute fold changes of miRNAs positively correlate with their p-values (Figure 1B, D), to select miRNAs likely to have decent effect sizes on cellular functions and to retain a reasonable power of detection, we ranked the miRNAs by their p-values and included those with p-values less than 0.1 for the miRNA-gene-GO enrichment analysis. This approach of using relaxed cutoff of p-values may give rise to more false positives than more stringent methods, such as multiple hypothesis testing, but the purpose of our bioinformatic analysis is to predict potential cellular outcomes of miRNA expression changes, hence some false positives are allowed. The TargetScan and Gene Ontology database were used in this analysis for miRNA target prediction and functional annotation of miRNA target genes respectively. To increase the power of bioinformatic analysis, we relaxed the p-value cutoff to 0.1 to include more miRNAs in the analysis. 29 (20 for 24 hours, 9 for 7 days) miRNAs had higher expression levels in the CCI samples than in the sham controls, and 39 (23 for 24 hours, 16 for 7 days) miRNAs showed reduced expression in the CCI group compared to sham controls (Table S2). We divided these miRNAs into 4 time- and change-direction defined test groups: 24 hour positive change group, 24 hour negative change group, 7 day positive change group and 7 day negative change group (Table S2). Each test group was analyzed independently.

**Figure 3:**
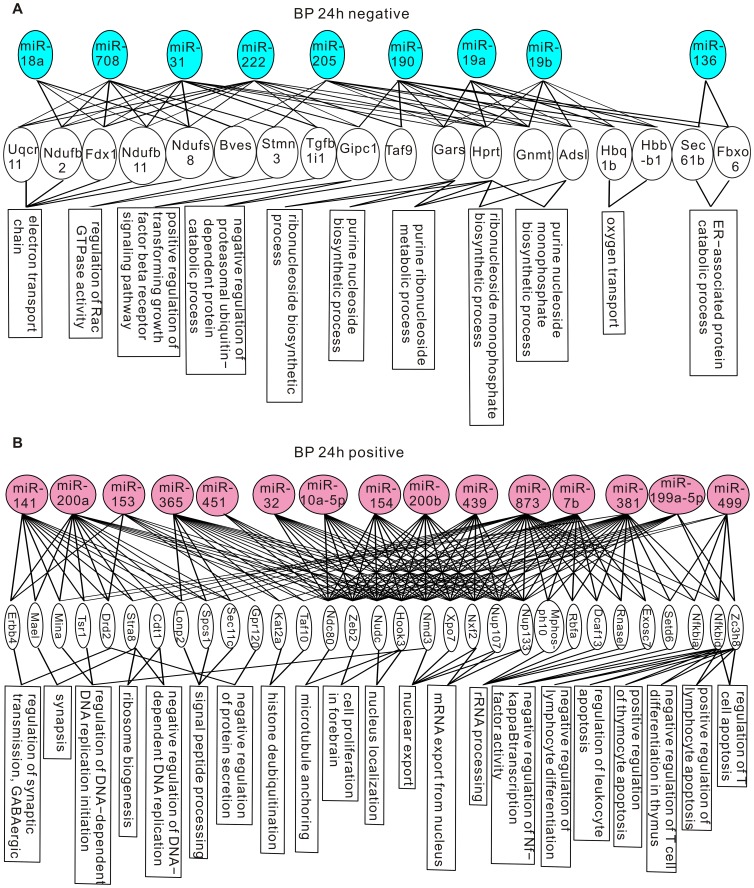
Genes and GO terms in the biological process ontology that are over-represented by differentially expressed miRNAs at 24 hours post-CCI. (A) Genes and GO terms enriched by down-regulated miRNAs. (B) Genes and GO terms enriched by up-regulated miRNAs.

**Figure 4:**
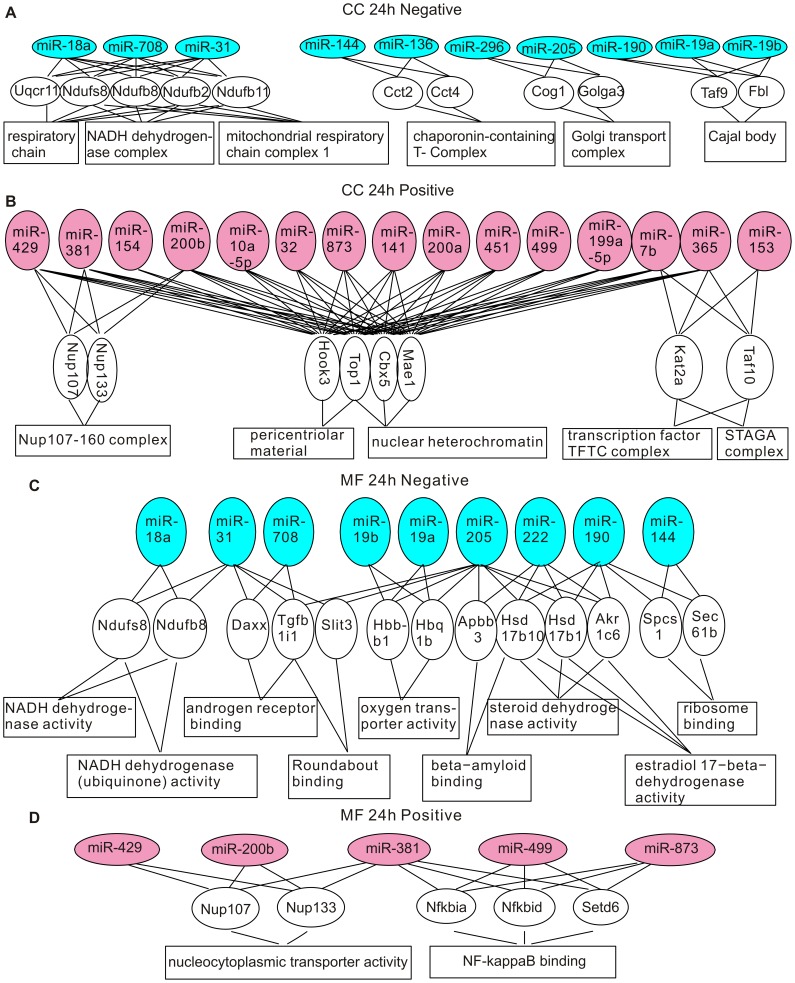
Genes and GO terms in the cellular component and the molecular function ontology that are over-represented by differentially expressed miRNAs at 24 hours post-CCI. (A) Genes and GO terms in the cellular component ontology enriched by down-regulated miRNAs. (B) Genes and GO terms in the cellular component ontology enriched by up-regulated miRNAs. (C) Genes and GO terms in the molecular function ontology enriched by down-regulated miRNAs. (D) Genes and GO terms in the molecular function ontology enriched by up-regulated miRNAs.

**Figure 5:**
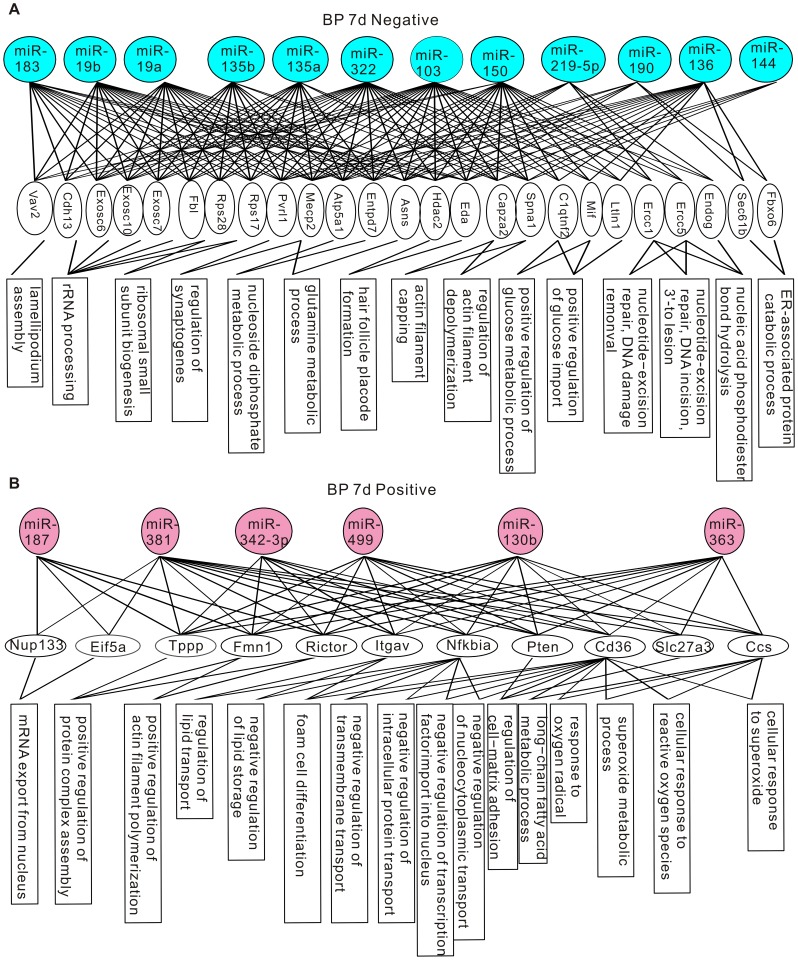
Genes and GO terms in the biological process ontology that are over-represented by differentially expressed miRNAs at 7 days post-CCI. (A) Genes and GO terms enriched by down-regulated miRNAs. (B) Genes and GO terms enriched by up-regulated miRNAs.

**Figure 6:**
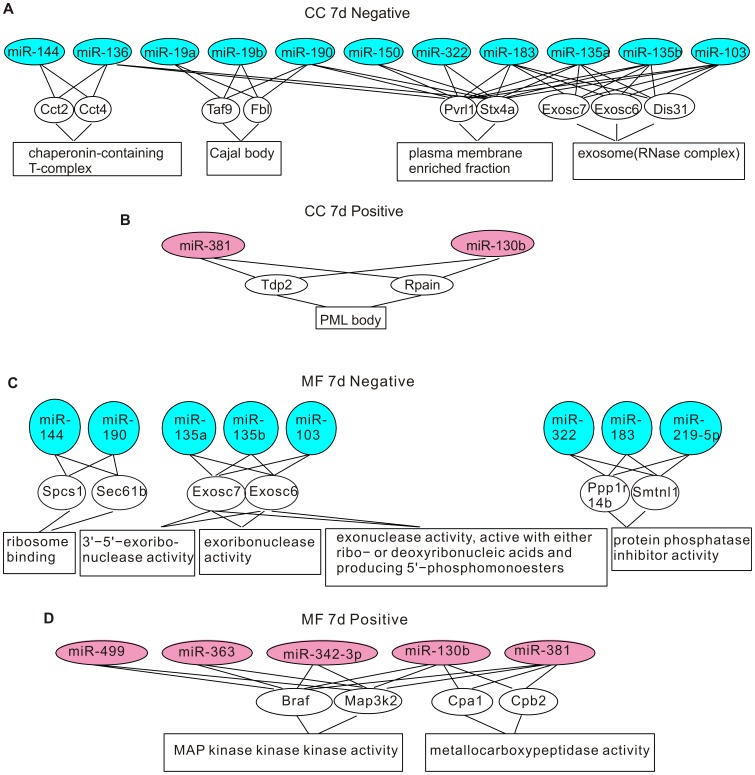
Genes and GO terms in the cellular component and the molecular function ontology that are over-represented by differentially expressed miRNAs at 7 days post-CCI. (A) Genes and GO terms in the cellular component ontology enriched by down-regulated miRNAs. (B) Genes and GO terms in the cellular component ontology enriched by up-regulated miRNAs. (C) Genes and GO terms in the molecular function ontology enriched by down-regulated miRNAs. (D) Genes and GO terms in the molecular function ontology enriched by up-regulated miRNAs.

The miRNA-gene-GO enrichment analysis was conducted in three steps. Because the rat genome is annotated less well than the mouse genome [23], to increase the coverage of genes, we used the mouse genome in our analysis. First, we matched the rat miRNAs identified by our deep sequencing to their mouse orthologs by using the TargetScan database (http://www.targetscan.org/mmu_50/mmu_50_data_download/Nonconserved_Family_Info.txt.zip). Next, in the miRNA-gene step, we collected all computationally predicted target genes of the mouse miRNA orthologs from the TargetScan database, and estimated their statistical significance of enrichment in each test group by using hypergeometric test. The genes that have p-values less than 10^−5^ in the enrichment test were considered over-represented. The number of over-represented genes for each test group is shown in Table S2. Lastly, in the gene-GO step, we obtained the GO terms that annotated the over-represented genes from the Gene Ontology database, and performed the enrichment analysis for each GO term by using conditional hypergeometric test. GO terms with p-values less than 0.05 were considered enriched. There were three sets of GO terms: biological process (BP), molecular function (MF), and cellular component (CC). The enriched GO terms of each category are shown in Figures 3, 4, 5, and 6.

Some identified GO terms have common cellular functions. For example, the GO-terms “mRNA export from nucleus”, “nuclear export” and “nucleus localization” are all related to nuclear transport. In Table S3, we grouped GO terms pointing to common functions together to obtain a generalized picture of the biological functions regulated by differentially expressed miRNAs. At 24 hours post-CCI, up-regulated miRNAs were enriched in cell proliferation and differentiation, chromatin remodeling, nuclear transport, protein synthesis and processing, synaptic transmission and white blood cell apoptosis (Table S3). miRNAs down-regulated at 24 hours post-CCI targeted to aerobic respiration, alternative splicing, protein maturation, protein catabolism, ribonucleoside metabolism, androgen signaling and intracellular signaling pathways (Table S3). Consistent with the overlapping but different miRNA expression patterns revealed by miRNA profiling, only intracellular signaling, protein maturation and alternative splicing were also targeted by miRNAs altered at 7 days post-CCI (Table S3). In addition to these functions conserved at both time points, at 7 days post-CCI, actin polymerization, cell-matrix adhesion, cellular response to ROS, intracellular trafficking, lipid metabolism and gene expression were targeted by up-regulated miRNAs, and down-regulated miRNAs were selective to actin depolymerization, alternative splicing, protein maturation, RNA degradation, nutrient metabolism, DNA repair and synaptogenesis (Table S3).

The GO enrichment and clustering analysis reveals that: 1. at the early times after brain injury, miRNAs might be involved in the inhibition of white blood cell apoptosis to augment the inflammatory response, the dampening of transcription and facilitation of protein folding and catabolism to reduce stress-induced denaturation and aggregation of intracellular proteins, and the promotion of aerobic respiration to exert protective actions; 2. At the later post-CCI times, miRNAs preferentially regulate gene expression, intracellular trafficking and metabolism, cytoskeleton and cell adhesion to allow cell structure remodeling and synaptogenesis for repair of neuronal circuitry. These findings suggest that miRNAs are involved in both the early post-CCI pathological changes and stress management, and the later brain repair mechanisms.

## Discussion

In this study, we examine the effect of CCI injury on the miRNA transcriptome in the hippocampus, and identify cellular functions and biological processes that are potentially regulated by miRNAs in the injured hippocampus. Our findings reveal that distinct sets of miRNAs are regulated at different post-CCI time points. To enquire the biological pathways targeted by the differentially-expressed miRNAs in a high-throughput manner and obtain a comprehensive view on the functional significance of miRNAs in TBI, we developed the miRNA-gene-GO enrichment analysis to functionally annotate miRNA expression changes. Our bioinformatic analysis shows that miRNAs target to multiple GO terms in a post-CCI-time-specific way. These findings indicate that miRNAs act in concert to regulate a broad range of cellular functions in the brain at different stages of TBI.

The fact that both up- and down-regulation of miRNAs are detected in the injured hippocampus indicates that the miRNA expression change is not caused by global changes of miRNA biogenesis or turnover. The observations that more miRNAs are altered at 24 hours than at 7 days post-CCI and that ∼75% of miRNAs changed at 24 hours restore their expression levels at 7 days suggest that the mechanisms to alter miRNA expression at various post-CCI times are different. In addition to the number, the identities of miRNAs altered at the two post-CCI time points that we examined are also different, thus each post-CCI stage has a unique miRNA expression profile, which can be potentially used as molecular markers for TBI progress. Redell and colleagues have used the microarray assay to examine miRNA expression changes at 3 hour and 24 hour after CCI [19]. The pool of differentially expressed miRNAs identified by their study does not overlap with our results. The discrepancy may be related to the difference in miRNA detection method (deep sequencing vs. microarray), and higher impact strength and older rats used in the Redell et al. study.

miRNA expression changes may be the outcome of injury-related pathological changes in the brain, such as cell death and neuroinflammation. If this is the case, we predict a uniform change direction for all or the majority of miRNA species, but only 6% of miRNAs detected in the hippocampus show altered expression, and both increases and decreases are observed, suggesting that miRNAs do not passively respond to injury. It is likely that modulating miRNA expression is an active approach taken by cells to cope with injury.

What is the consequence of miRNA expression changes? A miRNA usually has numerous mRNA targets and the effect of altering a single miRNA species on its targets may be moderate. The robustness of miRNA mediated gene expression regulation can be enhanced through cooperative action of several types of miRNAs that bind to the same target gene or to several genes serving for the same cellular process. We designed a miRNA-gene-GO enrichment analysis to test if distinct miRNAs changed in the same direction would also coordinately regulate cellular functions. miRNAs changed in the same direction have similar effects on their target expression, hence are put in the same group for the test. The results of our analysis show that many differentially expressed miRNAs are related to common GO terms. Both 24 hours and 7 days post-CCI correspond to the secondary damage stage of TBI, which occurs at minutes to days and even months after the initial trauma as the results of delayed histological, biochemical, metabolic and cellular changes [24], [25]. Studies using TBI animal models show that the increase of intracellular calcium resulting from altered balance of excitatory and inhibitory synaptic transmission triggers a cascade of events in the cell, such as reactive oxygen species production, calpain activation and mitochondrial damage [26], [27], [28]. These cellular responses are essential to the initiation of secondary damage [24], and are predicted to play important roles in TBI-related structural and functional impairment of neurons. The findings of our miRNA-gene-GO enrichment analysis are consistent with the known post-trauma-pathophysiological changes. For example, GO related to synaptic transmission, cellular response to ROS and aerobic respiration are targeted by miRNAs changed in CCI brains. In addition to the known cellular changes, our results show that many other cellular pathways, which are not recognized in previous TBI studies, are targeted by miRNAs in TBI brains.

The fact that distinct GO terms are identified for the two post-CCI time points is intriguing. At 24 hours post-CCI, miRNAs are modulated to inhibit leukocyte cell death for the inflammatory response, prevent stress-induced protein denaturation and aggregation, and protect cells from energy exhaustion. At the later recovery-oriented time, cells switch to miRNAs that bolster a permissive cellular environment for cell repair and structural remodeling. Hence, miRNA mediated gene expression regulation is an integral component of the cellular strategies to adapt to, compensate for and repair brain damages from traumatic brain injury. Future studies to understand the specific functions of each differentially expressed miRNA and the significance of the targeted cellular pathways for TBI may contribute to the amelioration of long-term cognitive dysfunction as a consequence of brain injury.

## Materials and Methods

### Controlled Cortical Impact (CCI) Injury

CCI injury was performed in male rats (Sprague Dawley, 200 g) as previously described [29], [30]. Briefly, rats were anesthetized with isoflurane and placed on a stereotaxic device. A 4.0 mm hole was made in the skull over the left tempoparietal cortex to expose the dura. Each animal received a single contusion (deformation depth: 2 mm; velocity: 3.5 m/sec; dwell time: 200 ms) applied through an impact tip (3 mm in diameter). Following CCI, the burr hole was covered with the skull cap, sealed with bone wax, and scalp sutured. Sham-operated control rats were operated similarly, but received no cortical impact.

### miRNA Sequencing Library Construction and Deep Sequencing

Dorsal hippocampi of rats were removed and homogenized with a Polytron homogenizer (Kinematica) in the lysis buffer provided by the mirVana miRNA Isolation Kit (Ambion). The lysate was then extracted with acid-phenol:chloroform, added ethanol to bring up the sample to 25% ethanol and fractionated to isolate the large and small RNAs by using the glass-fiber filter of mirVana miRNA Isolation Kit. miRNAs were purified from the small RNA fraction by using denaturing polyacrylamide gel electrophoresis (15%) followed by recovering the 17∼27-nt-long RNA fragments which were enriched for miRNAs. RNA integrity number (RIN) of the extracted RNA samples was measured to assess RNA quality by using the Agilent 2100 Bioanalyzer. Deep-sequencing libraries were constructed by using the Small RNA Sample Prep kit (Illumina). Briefly, 3′ adaptors and 5′ adaptors were ligated to purified miRNAs sequentially. The adaptor tagged miRNAs were reverse-transcribed and PCR amplified. The PCR products were purified by polyacrylamide gel electrophoresis (8%) and sequenced by using an Illumina Genome Analyzer II.

### Deep-sequencing Data Analysis

Sequence data analysis was performed as described previously [31]. Briefly, raw sequence reads were first consolidated by clustering identical sequence reads and only those containing complete 3′ and 5′ adaptor sequences were subjected to downstream analysis. After trimming of 3′ and 5′ adaptor sequences, the remaining sequences were aligned to the miRNA hairpin sequences downloaded from miRBase database release (http://www.mirbase.org/) by using Bowtie v0.2.13 [32]. The mappable reads were further filtered to remove those which did not map to mature miRNAs. The expression count of each type of miRNA was normalized to the total number of mapped reads of the corresponding sample [33]. Linear models, empirical Bayes methods [34] and Student’s t test were used for statistics.

### miRNA-gene-GO Enrichment Analysis

The following equation was used for the hypergeometric statistical test in the gene enrichment analysis for each test group (e.g. 24 h positive).



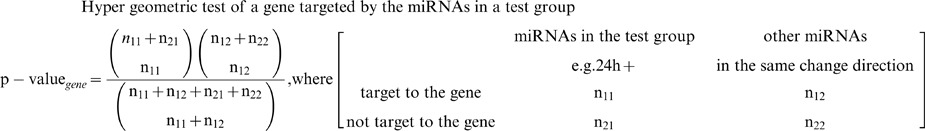
n_11_ =  the number of miRNAs in the test group that target to the gene being tested; n_12_ =  the number of other detected miRNAs that had the same change direction as those in the test group, and target to the gene being tested, but were not included in the test group because their p-values were greater than 0.1; n_21_ =  the number of miRNAs in the group that do NOT target to the gene being tested; n_22_ =  the number of other miRNAs that had the same change direction as those in the test group, but do NOT target to the gene being tested, and were not included in the test group because their p-values were greater than 0.1.

The enriched GO terms that annotate the over-represented genes were identified by using the conditional hypergeometric statistical test [R package GOStats, [35]]. The GO terms whose statistical significance was contributed by their significant children terms were removed in the conditional hypergeometric test. GO terms that had p-values less than 0.05 and were annotations for ≥2 over-represented genes that were targeted by ≥2 miRNAs in the test group were considered statistically enriched. The following equation was used for the test.
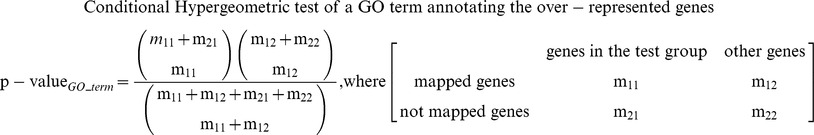
m_11_ =  the number of over-represented genes in the test group that were mapped to the GO term being tested; m_12_ =  the number of other genes in the GO database that were mapped to the GO term being tested; m_21_ =  the number of over-represented genes in the test group were NOT mapped to the GO term being tested; m_22_ =  the number of other genes in the GO database that were NOT mapped to the GO term being tested.

### qRT-PCR

miRNAs were extracted by using the mirVana miRNA Isolation Kit (Ambion). The TaqMan miRNA assay kit (Applied Biosystems) was used for qRT-PCR of miRNAs. Briefly, miRNAs were transcribed into cDNAs and amplified by PCR with the Taqman primers specific for miRNAs of interest by using the Applied Biosystems 7900 Fast Real-Time PCR System according to the manufacture’s manual. U6 was used as the endogenous control gene to normalize input amounts.

### Ethics Statement

This study was carried out in strict accordance with the recommendations in the Guide for the Care and Use of Laboratory Animals of the National Institutes of Health. The protocol was approved by Institutional Animal Care and Use Committee (IACUC) of Uniformed Services University of the Health Sciences (Permit Number: APG 09-748). All surgery was performed under isoflurane anesthesia, and all efforts were made to minimize suffering.

## Supporting Information

Figure S1 **P-value distribution for miRNAs detected in the rat hippocampus by deep-sequencing.** (A) Representative gel image of miRNA deep-sequencing libraries. (B) Frequency histogram of p-values for miRNAs analyzed at 24 hours post-CCI. (C) Frequency histogram of p-values for miRNAs analyzed at 7 days post-CCI.
(TIF)

Table S1 **RIN and summary of deep-sequencing reads.** The RIN, numbers of obtained sequence reads and mapped reads for each sample are listed.
(DOCX)

Table S2 **Summary of miRNA-gene-GO enrichment analysis.** The number of miRNAs in each test group and the number of genes over-expressed by each miRNA test group are listed.
(DOCX)

Table S3 **Clustering of GO terms.** GO terms enriched by each miRNA test group are classified into functional groups according to the biological functions they are associated with. Abbreviations: BP, biological process; CC, cellular component; MF, molecular function.
(DOCX)
