# Supplementary figures and images for: Expression of miRNAs and Their Cooperative Regulation of the Pathophysiology in Traumatic Brain Injury
Source: PLoS One. 2012 Jun 22;7(6):e39357. doi: 10.1371/journal.pone.0039357 (PMC3382215; doi:10.1371/journal.pone.0039357)

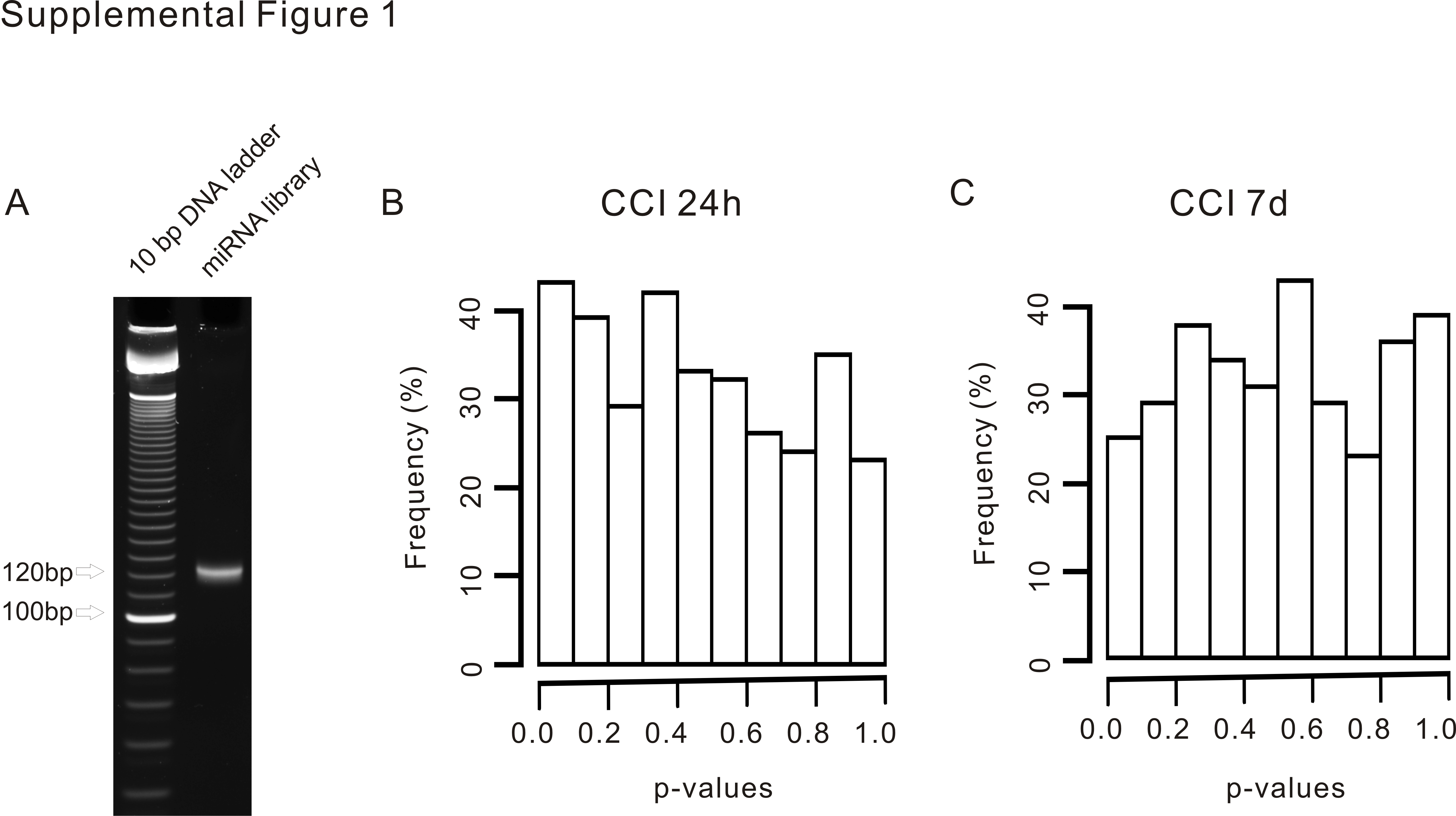

Supplement: Figure S1 — P-value distribution for miRNAs detected in the rat hippocampus by deep-sequencing. (A) Representative gel image of miRNA deep-sequencing libraries. (B) Frequency histogram of p-values for miRNAs analyzed at 24 hours post-CCI. (C) Frequency histogram of p-values for miRNAs analyzed at 7 days post-CCI. (TIF) [file pone.0039357.s001.tif]
