# Supplementary material for: Expression of miRNAs and Their Cooperative Regulation of the Pathophysiology in Traumatic Brain Injury
Source: PLoS One. 2012 Jun 22;7(6):e39357. doi: 10.1371/journal.pone.0039357 (PMC3382215; doi:10.1371/journal.pone.0039357)
Supplement: Table S1 — RIN and summary of deep-sequencing reads. The RIN, numbers of obtained sequence reads and mapped reads for each sample are listed. (DOCX) [file pone.0039357.s002.docx]

Supplemental Table 1

| Sample | RIN | # Reads | # Mapped | %Mapped |
| --- | --- | --- | --- | --- |
| CCI-24h-1 | 8.8 | 986568 | 890445 | 90 |
| CCI-24h-2 | 7.8 | 836330 | 771639 | 92 |
| CCI-24h-3 | 8.9 | 849061 | 781756 | 92 |
| CCI-24h-4 | 9.3 | 937142 | 854949 | 91 |
| Sham-24h-1 | 7.4 | 759777 | 679002 | 89 |
| Sham-24h-2 | 9.4 | 933243 | 867785 | 93 |
| Sham-24h-3 | 9.3 | 905133 | 840645 | 93 |
| CCI-7d-1 | 9.2 | 875319 | 808686 | 92 |
| CCI-7d-2 | 9.3 | 647090 | 587761 | 91 |
| CCI-7d-3 | 8.8 | 823094 | 763545 | 93 |
| CCI-7d-4 | 9.2 | 890554 | 817116 | 92 |
| Sham-7d-1 | 8.7 | 693583 | 628573 | 91 |
| Sham-7d-2 | 8.8 | 949861 | 876046 | 92 |
| Sham-7d-3 | 8.9 | 757987 | 686992 | 91 |
| Sham-7d-4 | 9.1 | 819822 | 763404 | 93 |
