# Supplementary material for: Expression of miRNAs and Their Cooperative Regulation of the Pathophysiology in Traumatic Brain Injury
Source: PLoS One. 2012 Jun 22;7(6):e39357. doi: 10.1371/journal.pone.0039357 (PMC3382215; doi:10.1371/journal.pone.0039357)
Supplement: Table S2 — Summary of miRNA-gene-GO enrichment analysis. The number of miRNAs in each test group and the number of genes over-expressed by each miRNA test group are listed. (DOCX) [file pone.0039357.s003.docx]

Supplemental Table 2

| Test group | Number of miRNAs | Number of over-represented genes |
| --- | --- | --- |
| CCI 24h negative | 23 | 5785 |
| CCI 24h positive | 20 | 3201 |
| CCI 7d negative | 16 | 4786 |
| CCI 7d positive | 9 | 2162 |
