# Supplementary material for: Expression of miRNAs and Their Cooperative Regulation of the Pathophysiology in Traumatic Brain Injury
Source: PLoS One. 2012 Jun 22;7(6):e39357. doi: 10.1371/journal.pone.0039357 (PMC3382215; doi:10.1371/journal.pone.0039357)
Supplement: Table S3 — Clustering of GO terms. GO terms enriched by each miRNA test group are classified into functional groups according to the biological functions they are associated with. Abbreviations: BP, biological process; CC, cellular component; MF, molecular function. (DOCX) [file pone.0039357.s004.docx]

Supplemental Table 3

**CCI 24 h Positive**

| Function | GO term |
| --- | --- |
| chromatin remodeling | nuclear heterochromatin (CC) |
|  | histone deubiquitination (BP) |
| cell proliferation | negative regulation of DNA-dependent DNA replication(BP) |
|  | regulation of DNA-dependent DNA replication initiation (BP) |
|  | cell proliferation in forebrain (BP) |
|  | Synapsis (BP) |
| cell differentiation | negative regulation of lymphocyte differentiation (BP) |
|  | negative regulation of T cell differentiation in thymus (BP) |
| microtubule anchoring | pericentriolar material(CC) |
| transcription | STAGA complex (CC) |
|  | transcription factor TFTC complex (CC) |
|  | negative regulation of NF-kappaB transcription factor activity (BP) |
|  | NF-kappaB binding (MF) |
| Nuclear transport | Nup107-160 complex (CC) |
|  | mRNA export from nucleus (BP) |
|  | nuclear export (BP) |
|  | nucleus localization (BP) |
|  | nucleocytoplasmic transporter activity (MF) |
| white blood cell apoptosis | positive regulation of lymphocyte apoptosis (BP) |
|  | positive regulation of thymocyte apoptosis (BP) |
|  | regulation of leukocyte apoptosis (BP) |
|  | regulation of T cell apoptosis (BP) |
| synaptic transmission | regulation of synaptic transmission, GABAergic (BP) |
| protein synthesis and processing | negative regulation of protein secretion (BP) |
|  | ribosome biogenesis (BP) |
|  | rRNA processing (BP) |
|  | signal peptide processing (BP) |

**CCI 24h Negative**

| Function | GO term |
| --- | --- |
| aerobic respiration | respiratory chain (CC) |
|  | mitochondrial respiratory chain complex I (CC) |
|  | NADH dehydrogenase complex (CC) |
|  | NADH dehydrogenase (ubiquinone) activity (MF) |
|  | NADH dehydrogenase activity (MF) |
|  | electron transport chain (BP) |
| protein maturation | chaperonin-containing T-complex (CC) |
|  | Golgi transport complex (CC) |
|  | ribosome binding (MF) |
| alternative splicing | Cajal body (CC) |
| androgen signaling | androgen receptor binding (MF) |
|  | estradiol 17-beta-dehydrogenase activity (MF) |
|  | steroid dehydrogenase activity (MF) |
| protein catabolism | ER-associated protein catabolic process (BP) |
|  | negative regulation of proteasomal ubiquitin-dependent protein catabolic process (BP) |
| intracellular signaling | regulation of Rac GTPase activity (BP) |
|  | positive regulation of transforming growth factor beta receptor signaling pathway (BP) |
|  | Roundabout binding (MF) |
|  | beta-amyloid binding (MF) |
| ribonucleoside  metabolism | purine nucleoside biosynthetic process (BP) |
|  | purine nucleoside monophosphate biosynthetic process (BP) |
|  | purine ribonucleoside metabolic process (BP) |
|  | ribonucleoside biosynthetic process (BP) |
|  | ribonucleoside monophosphate biosynthetic process (BP) |

**CCI 7d Positive**

| Function | GO term |
| --- | --- |
| intracellular trafficking | mRNA export from nucleus (BP) |
|  | negative regulation of transcription factor import into nucleus (BP) |
|  | negative regulation of nucleocytoplasmic transport (BP) |
|  | negative regulation of transmembrane transport (BP) |
|  | negative regulation of intracellular protein transport (BP) |
|  | regulation of lipid transport (BP) |
| gene expression | PML body (CC) |
|  | positive regulation of protein complex assembly (BP) |
| signaling | MAP kinase kinase kinase activity (MF) |
| cellular response to ROS | cellular response to reactive oxygen species (BP) |
|  | cellular response to superoxide (BP) |
|  | response to oxygen radical (BP) |
|  | superoxide metabolic process (BP) |
| lipid metabolism | negative regulation of lipid storage (BP) |
|  | long-chain fatty acid metabolic process (BP) |
| actin polymerization | positive regulation of actin filament polymerization (BP) |
| cell-matrix adhesion | regulation of cell-matrix adhesion (BP) |
|  | apoptotic cell clearance cd36,ITGAV, should be cell adhesion "cellular membrane organization" (BP) |

**CCI 7d Negative**

| Function | GO term |
| --- | --- |
| alternative splicing | Cajal body (CC) |
| actin depolymerization | actin filament capping (BP) |
|  | regulation of actin filament depolymerization (BP) |
| protein maturation | chaperonin-containing T-complex (CC) |
|  | ribosomal small subunit biogenesis (BP) |
|  | rRNA processing (BP) |
|  | ribosome binding (MF) |
| signaling | protein phosphatase inhibitor activity (MF) |
| DNA repair | nucleic acid phosphodiester bond hydrolysis (BP) |
|  | nucleotide-excision repair, DNA damage removal (BP) |
|  | nucleotide-excision repair, DNA incision, 3'-to lesion (BP) |
| nutrient metabolism | nucleoside diphosphate metabolic process (BP) |
|  | positive regulation of glucose import (BP) |
|  | positive regulation of glucose metabolic process (BP) |
|  | glutamine metabolic process (BP) |
|  | ER-associated protein catabolic process (BP) |
| RNA degradation | exosome (RNase complex) (CC) |
|  | 3'-5'-exoribonuclease activity (MF) |
|  | exonuclease activity, active with either ribo- or deoxyribonucleic acids and producing 5'-phosphomonoesters (MF) |
|  | exoribonuclease activity (MF) |
| synaptogenesis | plasma membrane enriched fraction (CC) |
|  | regulation of synaptogenesis (BP) |
|  | lamellipodium assembly (BP) |
